# Acute Effects of Whole-Body Vibration on Inflammatory Markers in People with Chronic Obstructive Pulmonary Disease: A Pilot Study

**DOI:** 10.1155/2018/5480214

**Published:** 2018-07-02

**Authors:** Vanessa K. S. Lage, Ana Cristina R. Lacerda, Camila D. C. Neves, Maria Gabriela A. Chaves, Aline A. Soares, Liliana P. Lima, Jeanne B. Martins, Mariana A. Matos, Érica L. M. Vieira, Antônio L. Teixeira, Hércules R. Leite, Vinicius Cunha Oliveira, Vanessa A. Mendonça

**Affiliations:** ^1^Programa Multicêntrico de Pós-Graduação em Ciências Fisiológicas, Sociedade Brasileira de Fisiologia, Universidade Federal dos Vales do Jequitinhonha e Mucuri, Diamantina, MG, Brazil; ^2^Laboratório de Inflamação e Metabolismo (LIM), CIPq Saúde, Universidade Federal dos Vales do Jequitinhonha e Mucuri, Diamantina, MG, Brazil; ^3^Programa de Pós-Graduação em Reabilitação e Desempenho Funcional, Universidade Federal dos Vales do Jequitinhonha e Mucuri, Diamantina, Minas Gerais, Brazil; ^4^Faculdade de Medicina, Universidade Federal de Minas Gerais, Belo Horizonte, Minas Gerais, Brazil

## Abstract

Whole-body vibration (WBV) has gained prominence in the rehabilitation of individuals with chronic obstructive pulmonary disease (COPD) because it is a safe and low intensity exercise that promises beneficial effects on physical performance and quality of life. However, its effects on plasma cytokine levels in COPD are still unclear. The aim of the current study was to investigate the acute effects of WBV on inflammatory biomarkers in people with COPD. Twenty-six participants, COPD people (n=13) and healthy controls (n=13), were included. Both groups performed WBV at amplitude of 2 mm and frequency of vibration of 35 Hz, during six series of 30 seconds. They were assessed for lung function, body composition, 6-minute walking test (6MWT), handgrip strength test, plasma concentrations of interleukin (IL), IL-6, IL-8, and IL-10, and soluble tumor necrosis factor alpha (TNF-*α*) receptors (sTNFR-1 and sTNFR-2). People with COPD had moderate disease [forced expiratory volume in the first second (FEV_1_) = 58.1%], as well as a worse performance in the 6MWT. The plasma cytokine profile at rest showed that participants with COPD had higher levels of IL-8 and lower levels of IL-10. After one session of WBV, we found an increased plasma IL-10 level in the COPD group, with similar levels for healthy controls. One session of WBV modified the plasma IL-10 level. No effects were found on the other investigated cytokines.

## 1. Introduction

Chronic obstructive pulmonary disease (COPD) is a respiratory disease characterized by chronic airflow obstruction associated with an inflammatory lung response to particles and/or toxics gases [1]. In this complex pulmonary inflammation process, there are inflammatory cells and mediators, such as cytokines tumor necrosis factor alpha (TNF-*α*), interleukin- (IL-) 8, IL-6, and IL-1*β* [2, 3].

Pulmonary inflammation markers released into the circulation are known as important factors explaining high cytokine levels in blood, characterizing a low-grade systemic inflammation [4]. A recent study of 14 inflammatory mediators (e.g., IL-10, IL-8, IL-6, and TNF-*α*) showed that most of the investigated cytokine levels in blood are higher for COPD when compared with those levels for smokers, former smokers without COPD, or nonsmokers [5]. This chronic systemic inflammation declines lung function and other patients' clinical and functional outcomes, such as quality of life and exercise tolerance [6]. At last, patients with persistent systemic inflammation have flare-ups and greater mortality rates [3, 7].

In this context, treatments for COPD should aim to decrease the low-grade systemic inflammation. Although previous studies have acknowledged that physical exercise may be effective on systemic inflammation related to COPD, which specific types of exercise, duration, and intensity have greater benefic effect sizes is still unclear [8, 9].

According to Brown et al. [10], intensity and duration of an exercise program have an important role in the systemic response to exercise. High-intensity exercises can lead to an inflammatory response through an interaction of inflammatory cells, hormones, cytokines, neural, and hematological factors [11]. It is speculated that these mediators are increased after high-intensity exercises because exercises can lead to muscle and connective tissue damage [12]. Previous studies supported this hypothesis. They found increased levels of IL-6, TNF-*α*, and transforming growth factor beta 1 (TGF-*β*1) after an acute exercise session [8, 9].

Whole-body vibration (WBV) emerges as an interesting physical exercise modality for patients with COPD because it is frequently classified as a mild stimulus demanding less muscle and cardiorespiratory effort than other modalities. Previous studies found benefic effects of WBV on muscle strength, physical performance, and quality of life for patients with COPD [13–15]. In addition, benefic effects of WBV were found on serum interleukin-8 levels and peroxisome-proliferator-activated receptor-*γ* coactivator 1*α* (PGC1-*α*) and irisin in patients with COPD, novel markers of muscle activity [16]. However, effects of WBV on systemic inflammation in COPD need further clarification. The aim of this study was to investigate the acute effects of WBV in inflammatory markers in COPD.

## 2. Materials and Methods

### 2.1. Participants

This was a pilot study including a convenience sample of 26 participants, thirteen participants with COPD and thirteen healthy controls, both sexes, recruited from the local community of Diamantina, in Brazil. To be included in the study, participants had to meet the following criteria: aged 45-80 years; no history of physical exercise in the last 3 months; no flare-up in the last 4 weeks; postmenopausal period for females; body mass index (BMI) < 30 kg/m^2^; no current usage of systemic corticosteroids; no severe comorbidities or self-reported contraindications for WBV (e.g., deep vein thrombosis, metal implants, pacemaker, epilepsy, tumors, arterial aneurysm, and arrhythmia). All participants were paired for sex, age, and BMI.

This study followed the Declaration of Helsinki [17] and obtained ethics approval from the Ethics and Research Committee of the Universidade Federal dos Vales do Jequitinhonha e Mucuri, Brazil (protocol 649.332). All participants consented to their participation in the project.

### 2.2. Assessment of Variables of Interest

Pulmonary function of all participants was assessed using spirometry, following the American Thoracic Society (ATS) and the European Respiratory Society (ERS) [18]. The classification of airflow limitation severity in participants with COPD was based on Global Initiative for Chronic Obstructive Lung Disease (GOLD) criteria [1]. According to post-bronchodilator forced expiratory volume in one second (FEV_1_), the degree of COPD group was classified as mild to very severe (GOLD I-IV).

Body composition (i.e., weight, lean mass, fat mass, fat percentage, and bone mineral density) was assessed by dual-energy X-ray absorptiometry (DXA, GE Lunar, Madison, Wisconsin, USA). BMI was calculated dividing the body mass by the square of the body height [19].

The history of smoking was assessed for smokers and former smokers using a self-report questionnaire. Number of pack-years was assessed, calculated as the number of smoked cigarettes per day/20, and multiplied by the number of years of smoking [20].

The exercise capacity was assessed using the six-minute walking test (i.e., 6MWT). The 6MWT was performed according to the guidelines of the ERS/ATS [18, 21] and the best out of two tests was analyzed.

The peripheral muscle strength was assessed using a hand dynamometer (SH5001, Saehan, Korea). Peak handgrip strength (KgF) was measured at the dominant side, with the elbow in a 90° flexion and the forearm and wrist in neutral position [22]. Three trials were performed, and the highest value was considered for analysis.

Blood samples were collected at rest and immediately after the intervention. The plasma cytokine levels (i.e., IL-6, IL-8, and IL-10) were measured using the cytometric bead arrays kit (BD Bioscience, San Jose, CA, USA), according to the manufacturer's protocol. Samples were acquired in a FACSCanto flow cytometer (BD Biosciences, San Jose, CA, USA) and analyzed using the FCAP array v1.0.1 software (Soft Flow Inc.). The detection limits were 1.6 pg/mL for IL-6, 1.2 pg/mL for IL-8, and 2.8 pg/mL for IL-10. Plasma soluble TNF-*α* receptor levels (i.e., sTNFR1, sTNFR2) were measured using conventional sandwich ELISA kits (DuoSet, R&D Systems, Minneapolis, MN, USA), according to the manufacturer's instructions. Detection limits were 5.0 pg/mL for kits.

### 2.3. Procedures

WBV was performed using a synchronous vibratory platform that produces vertical sinusoidal vibrations (*FitVibe, GymnaUniphy NV, Bilzen*, Belgium). Subjects exercised in a squatting position with 30° of knee flexion, with their feet 28 cm apart, barefoot, and with upper limbs holding the platform bars, performing six series of 30 seconds with 60 seconds of rest between each series. The vibratory stimulus was offered at an amplitude of 2 mm and a frequency of 35 Hz [14]. The protocol outlined above was based on previous studies [14, 23].

During the protocol, the oxygen consumption (VO_2_) was continuously monitored through open circuit spirometry by the K4b2 gas analyzer (COSMED), as well as heart rate (Polar FT7) and oxygen saturation (Nonin Onyx 9500). All participants had a preliminary session to familiarize with the vibrating platform and materials, avoiding any possible effect of anxiety, placebo, or attention control on physiological variables. This preliminary session was performed 24 hours before the intervention.

### 2.4. Statistical Analysis

Data were analyzed using the Graph-Pad Prism, version 5.0 (Inc., USA). Normality was tested using the Shapiro-Wilk test. Categorical variables were presented as absolute frequency, and these variables were compared using the chi-square test. Continuous variables were presented as means ± standard errors (SE), and these variables were compared using the independent* t*-test (for parametric distribution) and the Mann–Whitney test (for nonparametric distribution). Two-way ANOVA was used to analyze the interaction and effects of WBV on inflammatory variables, followed by post hoc* t*-test. Correlation between variables was performed using Pearson. The G power 3.1 software was used to calculate the power and effect size (i.e., Cohen's d). The significance level was p < 0.05.

## 3. Results

Characteristics of healthy and COPD participants are presented in Table 1. Participants with COPD showed had lower lung function and exercise capacity compared with healthy controls. According to the FEV_1_ values (post-bronchodilator) and the FEV_1_/forced vital capacity (FVC) ratio, participants with COPD were classified as moderate airway obstruction (GOLD 2) [1]. Moreover, participants with COPD had normal handgrip strength and worse smoking history when compared with controls.

About inhaled medication, 8 participants with COPD used bronchodilators and corticosteroids only. Although there are no significant differences in handgrip strength between groups, a moderate and significant inverse correlation was found between handgrip strength and IL-8 concentration (r= -0.43, p= 0.04) for participants with COPD.

Regarding the intensity of WBV, there was no difference in the oxygen consumption (p = 0.66, df = 22), with mean values of 6.5 (2.2) and 6.2 (1.1) ml.kg^−1^.min^−1^, between healthy and COPD participants, respectively. Thus, for both groups, the exercise was classified as “very light” intensity (<2 METS) according to the American College of Sports Medicine guidelines) [24].

**Figure 1:**
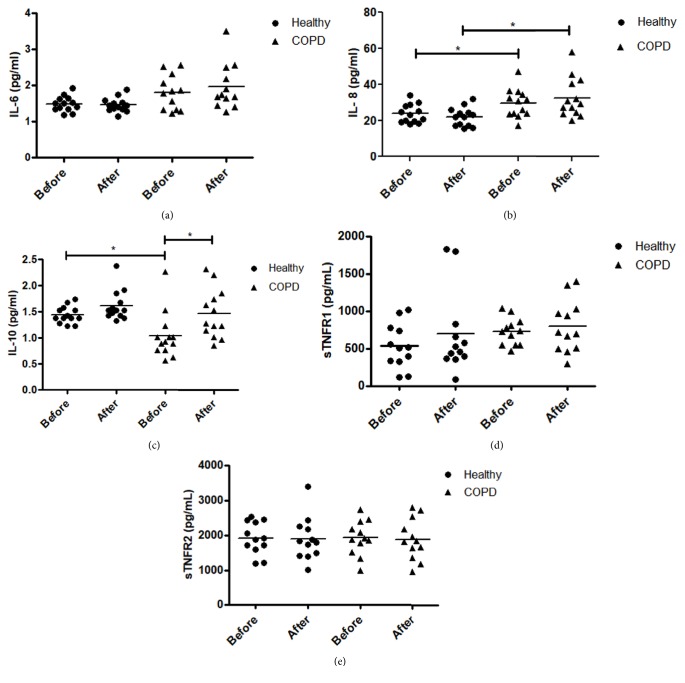
**Comparison of WBV for healthy and COPD participants on plasma levels of IL-6 (a), IL-8 (b), IL-10 (c), sTNFR-1 (d), and sTNFR-2 (e)**. Means ±SD. Two-way ANOVA and post hoc* t*-test. Difference between groups (*∗*) and difference between pre- and post-WBV (*∗∗*); COPD: chronic obstructive pulmonary disease; IL-6, IL-8, and IL-10: interleukin 6, interleukin 8, and interleukin 10; sTNFR1 and sTNFR2: soluble tumor necrosis factor receptors 1 and 2.

Plasma levels of IL-6, IL-10, IL-8, and soluble TNF-*α* receptors at rest and after WBV are shown in Figure 1. At rest, there was no difference between groups on plasma levels of IL-6 (p = 0.34), sTNFR-1 (p = 0.35), and sTNFR-2 (p = 0.98). However, participants with COPD showed higher IL-8 levels (p = 0.03, d = 0.87, power = 0.57, df = 24) and lower levels of IL-10 (p = 0.02; d = 1.21, power = 0.84, df = 24) compared with healthy controls.

After WBV, participants with COPD showed higher levels of IL-10 (p = 0.003, d = 0.94, power = 0.87, df = 24) compared with at rest, however, reaching values similar to values of healthy controls at rest (p = 0.89). In the COPD group, levels of IL-6, IL-8, sTNFR1, and sTNFR2 did not change after WBV [IL-6 (p = 0.08, df = 12); IL-8 (p = 0.25, df = 12); sTNFR-1 (p = 0.38, df = 12); and sTNFR-2 (p = 0.37, df = 12)]. Furthermore, after WBV, controls did not show significant differences in all analyzed markers (p> 0.05).

## 4. Discussion

The present study shows that stable COPD participants compared with healthy controls have different inflammatory rest state, with higher circulating levels of IL-8 and lower levels of IL-10. Furthermore, this study reported for the first time that WBV increases circulating levels of IL-10 in participants with COPD.

Systemic inflammation is considered a relevant characteristic and an important etiological factor of extrapulmonary manifestations in COPD [3]. Among inflammatory mediators, IL-8 may play a pivotal role. This cytokine is a member of the chemokine family and is a major chemoattractant to neutrophils, which are responsible for inducing and sustaining the inflammatory state of people with COPD. Against IL-8, IL-10 is a potent anti-inflammatory cytokine released from monocytes and alveolar macrophages in response to inflammatory stimuli [25].

In line with our results, previous studies have demonstrated that levels of IL-8 are increased in people with COPD [7, 26]. This increased level of proinflammatory markers in circulation seems to be related to clinical parameters impairments, such as quality of life and muscle strength [27–29]. Indeed, in the present study, levels of IL-8 were inversely associated with handgrip strength. Although the subjects of our study have shown no handgrip strength alterations, we believe that the maintenance of this proinflammatory state would be related to development of peripheral muscle dysfunction long term.

Few studies have investigated the role of IL-10 in the systemic inflammation of people with COPD. Similar to our results, recent studies have demonstrated that COPD people show reduced plasma levels [30, 31]. Interleukin-10 has been shown to suppress all the proinflammatory cytokines and is effective in blocking the synthesis of IL-8 [32]. Thus, we believe that the inflammatory status of participants in the present study, at least in part, is due to lower levels of IL-10. The mechanisms for this reduction are still unknown; however, it has been suggested that, as in asthma, macrophages from COPD people show a reduced production, which may help to amplify inflammation [25].

Although no between-group differences were found for sTNFRs and IL-6, previous studies found changes on these markers in people with COPD [33, 34]. One probable reason for this difference can be related to clinical characteristics of patients in our study. It has already been demonstrated that the extension of systemic inflammation in people with COPD is related to worse airflow limitation, lower body composition, and poor muscle strength [6, 7]. As noted, participants of this study showed moderate airflow limitation, stable disease, and BMI and muscle strength within the limits of normality. Thus, the severity of the disease in participants with DPOC of our study does not appear to be sufficient to promote changes in different inflammatory markers.

Physical exercise is the basis of the pulmonary rehabilitation of people with COPD. Although benefits are reported for the cardiovascular and muscular systems, exercise also stimulates proinflammatory cytokines, such as TNF-*α* and IL-6 [35]. According to Olfert* et al*. [36], it is well known that combination of exercise and high circulating inflammatory cytokine levels may bring more harm than benefit to people. In this context, we showed the effect of one session of WBV on systemic cytokines of people with COPD. Our results demonstrated that there was an increase in IL-10 levels in people with COPD, but the plasma levels of IL-6, IL-8, and soluble TNF-*α* receptors were not modified.

Studies investigating the acute effect of WBV in healthy people are scarce. Hazell* et al*. [37] reported that the addition of WBV to a dynamic exercise session (i.e., upper and lower body) resulted in no muscle damage as evidenced by no effects on muscle function or IL-1*β* and IL-6. However, IL-10 levels were higher after WBV. These authors suggested that the increase of IL-10 levels in the WBV group would have minimized any potential increase in other inflammation markers.

Several studies have investigated the response of cytokines to exercise in COPD people, but results are diverse. The main finding of Rabinovich* et al*. [38] was a consistent increase in plasma TNF-*α* levels after 11 min of moderate-intensity constant-work-rate cycling exercise in COPD people; this was not observed in healthy sedentary controls. Moreover, this phenomenon was not accompanied by changes in levels of sTNFRs or IL-6. Van Helvoort [39] demonstrated an increase on IL-6 response in people with muscle-wasted COPD after six-minute walking testing. Canavan* et al*. [40] showed that a maximal acute walking bout in COPD people does not change plasma IL-6 or TNF-*α* levels.

As revised by Brown* et al*. [10], the magnitude of the exercise stimulus, particularly duration and intensity, has often been related to important elements in stimulating IL-6 synthesis and secretion by skeletal muscles. Three out of the four studies that reported significant differences on IL-6 employed aerobic exercise protocols lasting approximately an hour. In this context, another study reported that 60 minutes of treadmill at 75% VO_2_ max compared with 55% and 65% VO_2_ max results in a greater increase on circulating IL-6 and IL-1ra levels, but not in TNF-*α* [41]. According to the authors, the higher respiratory quotient at 75% VO_2_ max supports the thesis that the exercise intensity-dependent effect on IL-6 might be related to a higher rate of carbohydrates utilization.

It has been showed that the strenuous exercise (marathon race) induces an increase in the proinflammatory cytokines TNF*α*, IL-1*β*, and IL-6. This is balanced by the release of cytokine inhibitors (IL-1ra, sTNFR1, and sTNFR2) and the anti-inflammatory cytokine IL-10. The study suggests that cytokine inhibitors and anti-inflammatory cytokines control magnitude and time of the inflammatory response to exercise [35]. Thus, increased production of anti-inflammatory cytokines during exercise may serve to restrict proinflammatory reactions to exercise-induced muscle damage [42], as well as associated with the development of disease states [43]. Thus, we can speculate that higher plasma levels of IL-10 after WBV could counterbalance a possible increase in IL-8 and IL-6.

Mechanisms by which WBV change IL-10 concentrations are still not well established; however, evidence indicates that glucocorticoids and catecholamines would be involved. These hormones stimulate the production of anti-inflammatory cytokines, such as IL-10 and IL-4 [44]. Although we have not evaluated hormonal parameters, there are studies that reported increased cortisol, for example, after WBV. Cardinale et al. [45] showed a significant increase on cortisol levels at the end of the vibration exercise (i.e., 30 Hz with 4-mm peak-to-peak displacement) compared with controls in the study in older people. Thus, we hypothesized that an acute session of WBV promoted the release of stress-related hormones that may have stimulated the release of IL-10 into the circulation. Future studies should investigate these mechanisms.

In the current literature, there is no consensus on the acute exercise-induced cytokine response. However, this is not very surprising because several factors are known to influence the acute response of exercise-induced cytokines, including body mass composition, age, and sex. The type, level, and time of exercise seem to influence outcomes [8, 44, 46].

Our data demonstrated (data not shown) that the WBV was performed at an intensity corresponding to about 50.9 to 53.5% of predicted heart rate (HR) max for age (220-years) in the healthy control group and 49.0 to 56.9% of the HR max in the COPD group. Thus, the exercise performed on the vibratory platform was characterized as “very light” (<2 METS) and may explain the absence of changes on these inflammatory parameters. Thus, the difference between our results and the actual literature could be partly explained by the low intensity of the exercise used in our protocol. This, in fact, is an interesting finding whereas although there was cardiorespiratory overload, the acute WBV session did not lead to an additional proinflammatory condition.

It is known that the adaptations of the various physiological systems to training result from the sum of the changes promoted by each individual exercise session. Thus, in the context of the possible benefits of vibration stimulus on inflammation parameters in COPD, it is crucial to discriminate acute and chronic vibration exercise responses [38]. Once the effect of a single WBV session in COPD is still poorly described in the literature, our findings may add to the current literature the understanding of the changes promoted by an acute WBV session on inflammatory profile providing important subsidies for the establishment of therapeutic strategies. As a perspective, the effect of WBV on inflammatory markers of COPD should be investigated.

The present study has some limitations that must be considered. The results cannot be extrapolated to other populations, since we do not know to what extent different diseases may interfere with the observed responses. The nonrandomized sample does not allow us to rule out the influence of selection bias and potential confounders. In order to avoid sample bias and study selection, groups were paired. In addition, future studies with greater samples evaluating additional inflammatory markers may allow a better understanding of WBV mechanisms in subjects with COPD.

## 5. Conclusions

Our data demonstrated that an acute session of WBV in people with COPD does not change levels of proinflammatory markers but was able to increase IL-10, an important anti-inflammatory marker. This trend needs to be addressed in further studies with greater samples. In addition, a better understanding of the acute effect of WBV on variables related to COPD will allow the elaboration of more effective training protocols and, consequently, this method may be an additional tool to complete rehabilitation programs.

## Figures and Tables

**Table 1:** Characteristics of participants (n=26).

Characteristics	Healthy controls n=13	COPD participants n=13	P-value
Sex (M/F)	9 /4	9 / 4	1.00^§^
Age (y)	63.4 ± 6.9	65.2 ± 7.6	0.52^¥^
BMI (kg/m^2^)	24.3 ± 2.6	22.6 ± 3.4	0.17^¥^
% Fat	28.9 ± 7.9	27.0 ± 7.8	0.53^¥^
Lean body mass (Kg)	41.7 ± 8.3	39.2 ± 8.4	0.44^¥^
Fat body mass (Kg)	16.9 ± 4.8	14.5 ± 4.3	0.19^¥^
Bone mass (g/cm^2^)	1.1 ± 0.1	1.0 ± 0.1	0.23^£^
FEV_1_ (l)	2.6 ± 0.3	1.5 ± 0.6	**<0.00**1^*∗¥*^
FVC (l)	3.4 ± 0.5	2.1 ± 0.8	**0.0**1^**∗***¥*^
FEV_1 postBD_ (% pred)	99.6 ± 12.5	58.1 ± 19.2	**<0.00**1^**∗***¥*^
FEV_1_/FVC (%)	76.9 ± 3.5	57.6 ± 9.1	**<0.00**1^**∗***¥*^
6MWT (m)	574.3 ± 18.8	445.7 ± 25.6	**<0.00**1^**∗***¥*^
Handgrip strength^#^ (KgF)	34.3 ± 2.4	34.8 ± 2.9	0.90^*¥*^
Pack-years (n)	12.5 ± 4.2	35.9 ± 6.3	**0.00**6^**∗***£*^

Means ±SD. ^§^Chi square test; ^¥^unpaired *t*-test; ^£^Mann–Whitney test; ^*∗*^statistical significance. BMI: body mass index; FEV_1_: forced expiratory volume in the first second; FVC: forced vital capacity; 6MWT: 6-minute walking test.# df=20.

## Data Availability

The data described in this article will be made available at any time when requested.
